# Quality evaluation of citrus varieties based on phytochemical profiles and nutritional properties

**DOI:** 10.3389/fnut.2023.1165841

**Published:** 2023-05-18

**Authors:** Huan Guo, Yin-Jian Zheng, Ding-Tao Wu, Xu Du, Hong Gao, Mutamed Ayyash, De-Guang Zeng, Hua-Bin Li, Hong-Yan Liu, Ren-You Gan

**Affiliations:** ^1^Research Center for Plants and Human Health, Institute of Urban Agriculture, Chinese Academy of Agricultural Sciences, National Agricultural Science and Technology Center, Chengdu, China; ^2^College of Biomass Science and Engineering and Healthy Food Evaluation Research Center, Sichuan University, Chengdu, China; ^3^Key Laboratory of Coarse Cereal Processing, Ministry of Agriculture and Rural Affairs, Sichuan Engineering and Technology Research Center of Coarse Cereal Industrialization, School of Food and Biological Engineering, Chengdu University, Chengdu, China; ^4^Department of Food Science, College of Agriculture and Veterinary Medicine, United Arab Emirates University, Al-Ain, United Arab Emirates; ^5^Pujiang Yuanxiang Modern Agriculture Limited Company, Chengdu, China; ^6^Guangdong Provincial Key Laboratory of Food, Nutrition and Health, Department of Nutrition, School of Public Health, Sun Yat-sen University, Guangzhou, China; ^7^Singapore Institute of Food and Biotechnology Innovation (SIFBI), Agency for Science, Technology and Research (A*STAR), Singapore, Singapore

**Keywords:** citrus, nutrients, flavonoids, phenolic acids, antioxidant activity

## Abstract

**Introduction:**

China is one of the major producers and exporters of various kinds of citrus fruits. As one of China’s major citrus planting bases, Sichuan has a citrus planting area that exceeds 400,000 hectares. Meanwhile, citrus cultivation has become one of the important agricultural pillar industries in the region. Citrus fruits are reported to show various health-promoting effects, especially antioxidant activity. However, reports on the functional, nutritional and qualitative characteristics of different citrus varieties in Sichuan are still scarce.

**Methods:**

The quality attributes (color parameters, shape, and size), juice properties (titratable acids and total soluble sugar), mineral elements, and health-promoting nutritional and functional components (protein, carbohydrates, fat, dietary fiber, ascorbic acid, phenolic acids, and flavonoids), as well as antioxidant properties of 10 typical citrus varieties cultivated in Sichuan, were systematically investigated and analyzed.

**Results and Discussion:**

Significant differences among different citrus varieties were found. In particular, the total soluble sugar content of Mingrijian was higher than that of other citrus, suggesting its potential for fresh consumption and food processing. Moreover, a total of five flavonoids and nine phenolic acids were identified and quantified. Yuanhong, with higher contents of ascorbic acid and phenolic acids, was considered to be a valuable variety with excellent antioxidant capacity and can be used for value-added processing in the food industry. Principal component analysis and hierarchical cluster heatmap analysis suggested that there were significant differences among the 10 citrus varieties. Correlation analysis confirmed the significant contribution of ascorbic acid and phenolic acids to antioxidant capacity in citrus. The results can provide some references for the cultivation and selection of nutritious citrus fruits.

## 1. Introduction

China is one of the major producers and exporters of various kinds of citrus fruits (*Rutaceae*), with an annual output of more than 30 million tons (1). The citrus family already has hundreds of natural or artificial cross-breeding varieties. Generally, there are three basic species of the *Citrus* genus, including pummelos (*Citrus maxima*), mandarins (*Citrus reticulata* Blanco), and citrons (*Citrus medica* L.). Until now, many hybrids have been developed based on these three species, including grapefruit (*Citrus paradisi* L.), lemon (*Citrus limon* L.), sour orange (*Citrus aurantium* L.), lime (*Citrus aurantifolia* L.), and sweet oranges (*Citrus sinensis* L.) (1–3). As one of the largest citrus planting bases in China, Sichuan is dominated by Chunjian, Buzhihuo, Daya, Shatangju, Wogan, etc. Relying on the unique natural geographical environment and advantageous citrus varieties, the annual production of citrus has exceeded 5 million tons in Sichuan (China Statistical Yearbook, 2022).^1^ Therefore, citrus has become one of the important agricultural pillar industries in the development of the Chengdu-Chongqing Area Twin-City Economic Circle, Southwestern China.

Citrus fruits are reported to show various health-promoting effects, especially antioxidant activity. Numerous studies have confirmed that a diet rich in antioxidants is directly associated with the prevention of a variety of human diseases, such as neurological disorders, diabetes, cardiovascular diseases, obesity, and several cancers (4–8). Citrus juice is the main citrus product on the market, and it is proposed to be a vital dietary source of various nutrients and bioactive compounds, including dietary fibers (e.g., cellulose and pectin), sugars (e.g., glucose, fructose, and sucrose), ascorbic acid, minerals (e.g., calcium, phosphorus, and potassium), carotenoids, flavonoids, flavanone glycosides (e.g., neohesperidin, hesperidin, and naringin), phenolic compounds (e.g., hydroxybenzoic acid and hydroxycinnamic acid), and volatile compounds (e.g., terpenes and esters) (9–12). In addition, citrus quality attributes, including color, seed, taste (sweetness and acidity), and flavor, are also of commercial importance (2). The contents of nutrients and bioactive components in different citrus varieties are different, and extensive studies have been carried out to isolate and characterize the chemical compounds of various citrus taxa. However, the phytochemical ingredients and antioxidant properties of some newly introduced citrus varieties (Yuanhong, Mingrijian, etc.) in China in recent years are still unknown. Assessing the phytochemical composition and biological properties of different citrus varieties is not easy, as it is influenced by many factors. It is well known that the types and contents of nutrients and bioactive ingredients in citrus fruits vary according to fruit maturity, cultivar., soil type, fertilizer applied, climate, and even different parts of the same fruit (2).

In order to provide a scientific basis for farmers to choose and plant citrus varieties with excellent nutritional quality, a comprehensive evaluation and comparison of the properties of citrus fruits are necessary. Therefore, the quality attributes, nutritional components, and functional components of 10 typical citrus varieties cultivated in Sichuan were systematically investigated and analyzed. This comparison was taken into account not only the easily assessable quality attributes (color parameters, shape, and size) and juice properties (titratable acids and total soluble sugar), mineral elements, and the health-promoting nutritional and functional components (protein, carbohydrates, fat, dietary fiber, ascorbic acid, phenolic acids, and flavonoids), as well as antioxidant properties of the citrus juice, were also included.

## 2. Materials and methods

### 2.1. Materials and chemicals

**Figure 1:**
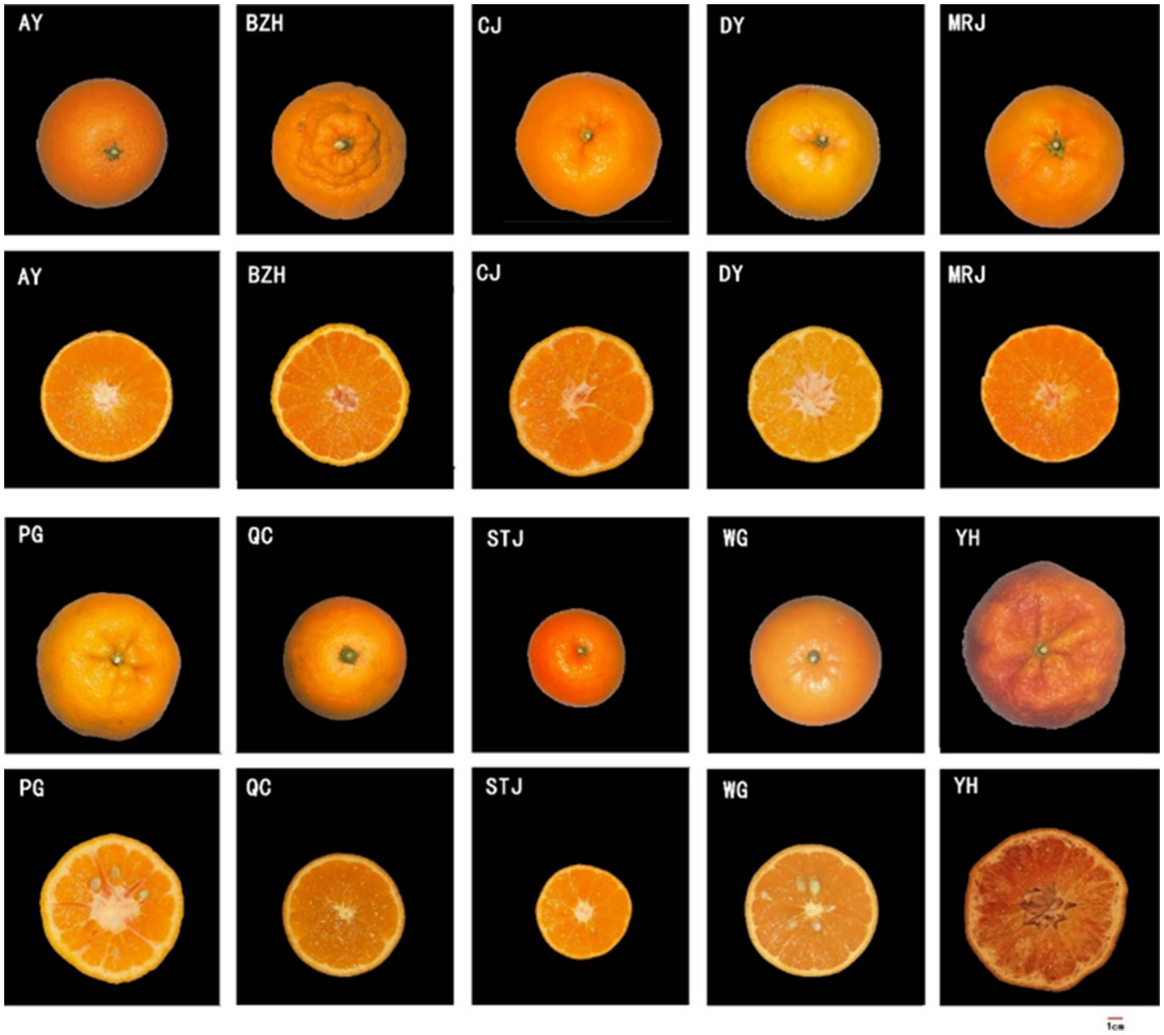
Appearance morphology of different citrus fruits. AY, Aiyuan 38; BZH, Buzhihuo; CJ, Chunjian; DY, Daya; MRJ, Mingrijian; PG, Ponkan; QC, Qicheng; STJ, Shatangju; WG, Wogan; YH, Yuanhong.

A total of 10 varieties of citrus (Figure 1) were studied, including Aiyuan 38 (*Citrus reticulata* Blanco ‘Aiyuan 38’), Buzhihuo (*Citrus reticulata* Blanco ‘Buzhihuo’), Chunjian (*Citrus reticulata* Blanco ‘Chunjian’), Daya (*Citrus reticulata* Blanco ‘Daya’), Mingrijian (*Citrus reticulata* Blanco ‘Mingrijian’), Ponkan (*Citrus reticulata* Blanco ‘Ponkan’), Qicheng (*Citrus sinensis* Osbeck ‘Qicheng’), Shatangju (*Citrus reticulata* Blanco ‘Shatangju’), Wogan (*Citrus reticulata* Blanco ‘Wogan’), and Yuanhong (*Citrus reticulata* Blanco ‘Yuanhong’). All samples were collected in Sichuan Province, China. Fruits were harvested at optimum maturity, carefully selected to ensure consistency in size and shape, and shipped to the laboratory immediately.

The 28 standards (> 95% purity), including 8-prenylnaringenin, cosmosiin, diosmin, didymin, hesperetin, xanthohumol, isosinensetin, poncirin, hesperidin, naringin, naringenin, nobiletin, sinensetin, tangeretin, neohesperidin, narirutin, quercetin, rutin, 4-hydroxybenzoic acid, chlorogenic acid, caffeic acid, protocatechuic acid, p-coumaric acid, gallic acid, vanillic acid, sinapic acid, salicylic acid, and syringic acid were obtained from Madsen Technology Co., Ltd. (Chengdu, China). Thermostable amylase, protease, and amyloglucosidase were obtained from Shanghai Anpu Experimental Technology Co., Ltd. (Shanghai, China). Ferric chloride, Dimethyl sulfoxide (DMSO), sodium acetate, 3-ethylbenzthiazoline-6-sulphonic acid (ABTS), 6-hydroxy-2,2-2,5,7,8-tetramethylchromane-2-carboxylic acid (Trolox), 2,2-diphenyl-1-picrylhydrazyl (DPPH), and 2,4,6-tripyridyl-s-triazine (TPTZ) were purchased from Beijing Solarbio Science and Technology Co., Ltd. (Beijing, China). The HPLC-grade acetonitrile and HPLC-grade phosphoric acid were purchased from Shanghai Macklin Biochemical Co., Ltd. (Shanghai, China). Besides, all the other chemical reagents were of analytical grade.

### 2.2. Determination of quality attributes, total soluble sugar, and titratable acids in citrus fruits

The quality attributes, total soluble sugar, and titratable acids of citrus were conducted according to the previous report (3). Color parameters (L*, a*, and b*) of citrus fruits were carried out using a spectrophotometer (YS3010, Shenzhen 3nh Technology Co., Ltd., Shenzhen, China). L* represents the lightness of color, a* represents the red/green chromatic coordinate, and b* represents the blue/yellow chromatic coordinate. Four evenly distributed equatorial points were measured for each citrus, and the average value was calculated from the measurements of ten fruits selected for each variety. The fruit shape index (d/h) was calculated by dividing the transverse diameter by the vertical length of the fruit. After being peeled and deseeded, the fruit samples were weighed and then the edible proportion (%) was calculated as the percentage of the fresh weight of the edible tissues to the weight of the whole fruit. Total soluble sugar (TSS) was measured with a hand-held digital refractometer (precision of ±0.01, PAL-BX/ACID F5, ATAGO Co., Ltd., Tokyo, Japan) at 25°C, and the data was expressed as “°Brix.” The refractometer was cleaned with distilled water after each measurement. Titratable acids (TA) were measured using the Phenolphthalein indicator method according to Li et al. (13). The samples were titrated with 0.1 M NaOH solution and the data was expressed as “%.”

### 2.3. Determination of the contents of main nutritional compositions in citrus fruits

After the fruit was washed, the flesh and peel portions were separated by hand. The flesh portion was chopped, and homogenized using a Polytron blender (MJ-WBL2521H, Midea Group Co., Ltd., Foshan, China) for 1 min. Afterward, a weighed portion (100 g) was oven-dried and finely ground for further nutritional analysis. The content of moisture was determined using the hot-air drying method. Ash, protein, fat, and crude fiber were determined according to previously reported methods (14). Briefly, the fruit sample (1.0 g) was placed in a muffle furnace (SXL-1008, Jinghong Experimental Equipment Co., Ltd., Shanghai, China). The furnace temperature was gradually raised to 550°C and maintained for 30 min. After cooling the sample for 30 min, the furnace temperature was slowly raised to 550°C again and maintained for 30 min until the ash sample was obtained with a constant weight. The protein content (nitrogen × 6.25) was estimated by the Kjeldahl method, and a nitrogen analyzer (KDN-103F, Shanghai Xianjian Instrument Co., Ltd., Shanghai, China) was applied. Total dietary fiber content was measured by enzymatic digestion with thermostable 50 μL amylase (300 U), 100 μL protease (30 U), and 100 μL amyloglucosidase (400 U) in 40 mL 4-Morpholineethanesulfonic acid-Tris buffer (MES-Tris, pH 8.2). The fat content was measured using petroleum ether as the extractant in a Soxhlet apparatus, and the carbohydrate content was measured by the difference method (15). The carbohydrate content was estimated according to the formula: carbohydrate (%) = 100 − ash−moisture−fat−protein−dietary fiber. Moreover, the content of ascorbic acid was determined according to a previous report (16). Briefly, citrus samples (100 g) were weighed and mixed with 100 mL of the extractant solution (8% acetic acid and 3% metaphosphoric acid). Thymol blue was selected as the indicator, and the fluorescence intensity was measured at an emission wavelength of 350 nm and an excitation wavelength of 430 nm. All nutritional compositions were presented in wet weight.

### 2.4. Determination of the mineral elements in citrus fruits

The fluoride (F) element was quantified by a fluoride ion-selective electrode (ISE) according to a previous study with appropriate modifications (17). Briefly, 1.0 g of the sample was weighed and placed in a volumetric flask (50 mL), and 10 mL of hydrochloric acid was added and reacted for one hour. Then, 25 mL of total ionic strength adjustment buffers were added and the volume was made up with distilled water. Connect the fluoride ion electrode (PF-2-01, Shanghai INESA Scientific Instrument Co., Ltd., Shanghai, China) and the calomel electrode (232–01, Shanghai INESA Scientific Instrument Co., Ltd., Shanghai, China) to the positive and negative electrodes of the measuring instrument, respectively. Then, the electrode was inserted into a 50 mL polyethylene plastic bottle filled with water, the magnetic stirrer was turned on at the same time, and the measurement was performed after the potential was equilibrated. Standards were determined at concentrations of 0.01, 0.02, 0.04, 0.1, and 0.2 μg/mL.

Macro elements, such as phosphorus (P), potassium (K), magnesium (Mg), calcium (Ca), and sodium (Na), as well as trace elements such as iron (Fe) and zinc (Zn), were analyzed using an inductively coupled plasma-optical emission spectrometry (ICP-OES; Varian ICP 720-ES, Varian Inc., Palo Alto, CA, United States) according to a previous study with appropriate modifications (18). Briefly, the sample (1.0 g) was added with 1 mL of HClO_4_ and 5 mL of HNO_3_ and treated on a hot plate. Where necessary, more acid was added to facilitate the dissolution of the residue, and a colorless and transparent digestion solution was prepared. Finally, the digested samples were cooled, fixed to volume, and filtered. Blanks were prepared in the same way as samples. The ICP-OES was calibrated using standard solutions of various elements prior to the analysis of citrus samples.

Trace elements, namely manganese (Mn), copper (Cu), and selenium (Se), were quantified using inductively coupled plasma-mass spectrometry (ICP-MS; Agilent 7,900, Santa Clara, CA, USA). A microwave-assisted digestion procedure was conducted using a microwave digester (Jiguang-6, Shanghai Yiyao Instrument Technology Development Co., Ltd., Shanghai, China) according to the method described by Hong et al. (19) with slight modifications. Briefly, 0.5 g of the sample was weighed and placed in a 100 mL flask. Then, 6 mL of a freshly prepared mixture of concentrated HNO_3_–H_2_O_2_ (5:1, *v/v*) was added, and the mixture stood for 10 min. Finally, digestion was performed in the microwave system under the following conditions: 120°C for 5 min, vent for 5 min, 150°C for 5 min, vent for 10 min, 190°C for 5 min, and vent for 20 min. After cooling, the resulting mixtures were fixed to 10 mL with 1 M HNO_3_. Blank samples were treated in the same way as the citrus samples.

### 2.5. HPLC analysis of phenolic acids and flavonoids

Citrus juice samples were obtained by manually squeezing the citrus fruit. The juice was stored at −20°C before analysis. The samples were thawed and centrifuged at 10,000× *g* for 10 min at room temperature to remove solids from the juice. The juice supernatant was used for HPLC analyses. The phenolic acids and flavonoids in citrus juice were identified and quantified using an Agilent 1,260 II HPLC (Agilent, Santa Clara, CA). The instrument was equipped with a quaternary pump, column thermostat, in-line degasser, autosampler, and DAD detector. An Agilent Zorbax SB-C18 column (4.6 × 150 mm, 5 μm) was used at 30°C. Water containing 0.1% phosphoric acid (solvent A) and acetonitrile (solvent B) constituted the mobile phase. The gradient and time of the mobile phase were adjusted to provide optimal separation of the major phenolic acids and flavonoids present. The gradient program was as follows: 20 ~ 30% B, 0–3 min; 30 ~ 40% B, 3–10 min; 40 ~ 50% B, 10–17 min; 50 ~ 60% B, 17–19 min; 60 ~ 70% B, 19–21 min; 70 ~ 80% B, 21–25 min; and 80 ~ 90% B, 25–30 min. Citrus juice was diluted 4 times with ultrapure water and filtered through a 0.22 μm filter before injection. The fixed flow rate and injection volume were 0.5 mL/min and 10 μL, respectively. The contents of phenolic acids and flavonoids in citrus juice were expressed as “μg/mL.”

The optimal detection wavelength for each standard was determined by comparing the peak areas of the different standards at the detection wavelengths of 255, 284, 336, and 370 nm. According to the test results, rutin, 4-hydroxybenzoic acid, protocatechuic acid, and vanillic acid were quantified at a wavelength of 255 nm; 8-prenylnaringenin, hesperetin, poncirin, naringin, hesperidin, neohesperidin, didymin, narirutin, naringenin, p-coumaric acid, gallic acid, salicylic acid, and syringic acid were quantified at a wavelength of 284 nm; cosmosiin, diosmin, isosinensetin, sinensetin, nobiletin, tangeretin, chlorogenic acid, caffeic acid, and sinapic acid were quantified at a wavelength of 336 nm; and xanthohumol and quercetin were quantified at a wavelength of 370 nm.

### 2.6. Determination of antioxidant activities

The antioxidant activities of citrus juice were determined by the ferric reducing antioxidant power (FRAP assay), ABTS cation radical, and DPPH radical scavenging activity according to previous reports (20, 21). The sample extraction and dilution methods were performed according to a previous study with slight modifications (4). Briefly, the fresh-squeezed citrus juice was diluted 1:1 (*v/v*) with methanol and extracted with stirring for 60 min. Then, it was centrifuged at 5000× *g* for 10 min. Before the measurement, the supernatant was collected and diluted 6, 7, 8, 9, and 10 times with 50% of methanol.

### 2.7. Statistical analysis

All experiments were carried out in triplicate, and the data were presented as means ± standard deviation. IBM SPSS Statistics 20 software (IBM, New York, USA) was used to analyze the data on fruit quality, nutritional composition, HPLC results, and antioxidant capacities. A one-way analysis of variance (ANOVA) plus *post hoc* Duncan’s test was performed to analyze the differences among different citrus samples for quality, composition, and antioxidant capacity. Moreover, origin 2021b software (OriginLab Inc., Northampton, MA, USA) was employed to conduct principal component analysis (PCA), hierarchical cluster heatmap analysis, correlation analysis, discrimination analysis and their plots.

## 3. Results

### 3.1. Analysis of citrus fruit quality attributes, total soluble sugar, and titratable acids

The quality attributes (color parameters, fruit weight, and edible proportion) and juice properties (TSS, TA, and TSS/TA) of citrus fruits are shown in Table 1. The appearance and cross-section photographs of different citrus varieties were displayed in Figure 1. The mean fruit weight, was a significant difference among different citrus varieties. STJ, a relatively small variety, had the lowest mean fruit weight (80.54 ± 11.29 g). However, no significant difference was found in fruit shape indexes among different varieties. Color parameters were important factors that affected the appearance of citrus and consumers’ decisions. As shown in Table 1, there were differences in the chroma L* and color index (a* and b*) values among different varieties of citrus juices. In particular, YH exhibited a darker red color, as indicated by the results of color measurements. Conversely, other citrus fruits exhibited a brighter orange color. In addition, the edible proportion varied greatly among all citrus, where MRJ achieved the highest edible proportion (87.79 ± 2.15%), while PG (68.33 ± 5.37%) and YH (70.13 ± 4.72%) had the lowest edible proportion. The edible proportion of citrus fruits was primarily determined by the number of seeds and peel thickness. The TSS, TA, and TSS/TA were also important parameters, related to citrus quality, where MRJ had the highest TSS/TA value (10.12), and QC had the lowest TSS/TA value (1.71). According to the above results, noticeable differences were observed among citrus varieties. As discussed in literature reports (22, 23), the quality parameters of citrus samples varied with varieties and growing regions. Notably, among the selected citrus varieties, the MRJ was a new variety introduced from Japan in 2011, and higher attention was given to it. The TSS/TA value of MRJ produced in Meishan, Sichuan was higher than other varieties, even higher than STJ (a variety famous for its high sweetness), indicating its potential for fresh consumption and food industrial processing.

**Table 1:** Quality attributes, total soluble sugar, and titratable acids of citrus fruits.

Varieties (Abbreviation)	Origin	Weight (g)	Seed	Edible proportion (%)	Fruit shape index (d/h)	Color parameters			TA (%)	TSS (°Brix)	TSS/TA ratio
						L*	a*	b*			
STJ	Meishan City, Sichuan Province	80.54 ± 11.29^a^	5 ± 2	72.87 ± 2.73^ab^	1.19 ± 0.12^ab^	16.76 ± 1.35^cd^	5.33 ± 0.84^a^	28.45 ± 2.46^de^	2.39 ± 0.13^b^	15.9 ± 1.2^f^	6.65
DY	Meishan City, Sichuan Province	228.47 ± 17.62^d^	N.A.	82.12 ± 3.29^cd^	1.18 ± 0.11 ^ab^	21.58 ± 2.04^e^	4.80 ± 0.67^a^	21.51 ± 3.18^bc^	3.06 ± 0.21^de^	13.2 ± 0.4^cd^	4.31
AY	Meishan City, Sichuan Province	182.72 ± 21.39^b^	N.A.	85.66 ± 3.02^d^	1.12 ± 0.15 ^ab^	14.25 ± 2.66^bc^	5.07 ± 0.92^a^	24.00 ± 2.83^bcd^	3.24 ± 0.26^e^	14.8 ± 0.6^e^	4.57
PG	Zizhong City, Sichuan Province	167.93 ± 10.75^b^	11 ± 5	68.33 ± 5.37 ^a^	1.29 ± 0.15^b^	20.48 ± 2.93^e^	5.51 ± 1.43^a^	25.79 ± 2.26^cd^	2.42 ± 0.18^bc^	11.9 ± 0.5^b^	4.92
QC	Chengdu City, Sichuan Province	164.75 ± 12.88^b^	N.A.	79.20 ± 3.82^bc^	1.01 ± 0.09 ^a^	19.34 ± 1.82^de^	3.85 ± 0.45^a^	20.27 ± 2.55^b^	7.47 ± 0.37^f^	12.8 ± 0.5^bc^	1.71
WG	Chengdu City, Sichuan Province	192.64 ± 15.10^bc^	9 ± 4	78.53 ± 3.56^bc^	1.19 ± 0.13 ^ab^	19.33 ± 1.89^de^	4.55 ± 1.05^a^	33.28 ± 3.27^e^	2.99 ± 0.19^de^	14.1 ± 0.5^de^	4.72
MRJ	Meishan City, Sichuan Province	178.35 ± 15.93^b^	N.A.	87.79 ± 2.15^d^	1.23 ± 0.15 ^ab^	14.70 ± 1.25^bc^	4.83 ± 1.66^a^	29.01 ± 3.60^de^	1.68 ± 0.25^a^	17.3 ± 0.6^g^	10.30
CJ	Pujiang City, Sichuan Province	237.19 ± 16.55^de^	1 ± 0	78.01 ± 3.09 ^bc^	1.15 ± 0.12 ^ab^	12.41 ± 1.31^b^	3.37 ± 0.77^a^	20.58 ± 2.62^b^	2.75 ± 0.22^bcd^	13.1 ± 0.4^cd^	4.76
BZH	Pujiang City, Sichuan Province	258.43 ± 12.16^e^	3 ± 1	78.44 ± 2.99 ^bc^	1.02 ± 0.18^a^	15.74 ± 1.46^bc^	4.16 ± 1.29^a^	26.93 ± 2.88^d^	2.83 ± 0.17^cde^	13.8 ± 0.6^cde^	4.88
YH	Meishan City, Sichuan Province	211.79 ± 20.74^cd^	N.A.	70.13 ± 4.72^a^	1.27 ± 0.13 ^ab^	9.14 ± 0.83^a^	21.96 ± 2.62^b^	15.11 ± 1.64^a^	2.85 ± 0.23^de^	10.8 ± 0.4^a^	3.79

Values represent mean ± standard deviation, and statistical analysis was carried out by ANOVA plus *post hoc* Ducan’s test, and statistical significance (*p* < 0.05) was indicated with different letters (a–e). AY, Aiyuan 38; BZH, Buzhihuo; CJ, Chunjian; DY, Daya; MRJ, Mingrijian; PG, Ponkan; QC, Qicheng; STJ, Shatangju; WG, Wogan; YH, Yuanhong; TA, titratable acids; TSS, total soluble sugar.

### 3.2. Nutritional composition analysis

The health-promoting effects of citrus fruits are largely attributed to their abundance of nutrients. Citrus fruits are rich in a variety of other nutritional compositions, including protein, carbohydrates, fat, dietary fiber, and ascorbic acid. The nutritional compositions of different citrus varieties were shown in Table 2. The total lipid content of citrus fruits was determined to be 0.42–1.31 g/100 g wet weight. The carbohydrate content of citrus fruits was determined to be 8.22–11.22 g/100 g wet weight and was the main component that gives citrus its sweetness (2). The dietary fiber of the citrus fruits varied significantly among different varieties, ranging from 2.20 to 4.28 g/100 g wet weight. YH was the variety with the highest dietary fiber content. BZH also had elevated dietary fiber content at about 4.03 g/100 g wet weight, while STJ (2.36 g/100 g wet weight) and WG (2.20 g/100 g wet weight) had the lowest dietary fiber content. As shown in Table 2, QC (39.26 ± 0.62 mg/mL) and BZH (38.17 ± 0.57 mg/mL) had the highest ascorbic acid content with no statistically significant difference, followed by YH (34.08 ± 0.65 mg/mL) and MRJ (33.92 ± 0.54 mg/mL). Furthermore, the lowest ascorbic acid content was found in WG (26.89 ± 0.37 mg/mL) and DY (23.28 ± 0.27 mg/mL). Regarding the ascorbic acid content, our results were in agreement with those reported by Cano et al. (11), i.e., orange varieties had a higher content of ascorbic acid compared to mandarin varieties. In addition, we found that the ascorbic acid content in QC was highest among all citrus varieties, and the same was true for the titratable acids. A correlation between the titratable acids and ascorbic acid content in citrus juice appears to be found, which is consistent with previously reported results (24). Regrettably, the taste of QC with high ascorbic acid content does not seem to be satisfactory due to the lowest value of TSS/TA. Differences in chemical composition were found in the varieties studied and could be attributed to environmental, physiological, and genetic factors (11).

**Table 2:** Nutritional composition and elemental composition of citrus fruit.

Varieties	STJ	DY	AY	PG	QC	WG	MRJ	CJ	BZH	YH
Protein (g/100 g wet weight)	0.91 ± 0.04 ^b^	1.23 ± 0.06 ^d^	1.39 ± 0.05^e^	1.34 ± 0.07^e^	1.02 ± 0.05^c^	1.21 ± 0.04^d^	0.82 ± 0.04 ^a^	0.79 ± 0.05^a^	0.94 ± 0.05^b^	0.80 ± 0.03^a^
Fat (g/100 g wet weight)	0.65 ± 0.04^b^	0.69 ± 0.03^c^	0.78 ± 0.03^c^	1.31 ± 0.03^f^	0.88 ± 0.04^d^	0.42 ± 0.02^a^	0.72 ± 0.02^c^	0.93 ± 0.04^e^	0.87 ± 0.04^d^	0.73 ± 0.03^c^
Carbohydrate (g/100 g wet weight)	10.34 ± 0.14^e^	8.22 ± 0.11^a^	10.05 ± 0.16^d^	8.92 ± 0.14^b^	8.88 ± 0.12^b^	8.37 ± 0.10^a^	9.62 ± 0.13^c^	10.57 ± 0.15^f^	8.90 ± 0.12^b^	11.22 ± 0.18^g^
Dietary fiber (g/100 g wet weight)	2.36 ± 0.05^b^	2.62 ± 0.06^c^	2.69 ± 0.06^c^	3.35 ± 0.07^d^	3.43 ± 0.08^e^	2.20 ± 0.06^a^	3.59 ± 0.06^f^	3.62 ± 0.06^f^	4.03 ± 0.08^g^	4.28 ± 0.09^h^
Ascorbic acid (mg/100 g wet weight)	30.55 ± 0.47^c^	23.28 ± 0.27^a^	31.11 ± 0.45^d^	31.94 ± 0.44^e^	39.26 ± 0.62^h^	26.89 ± 0.37^b^	33.92 ± 0.54^f^	31.38 ± 0.60^d^	38.17 ± 0.57^g^	34.08 ± 0.65^f^
Na (mg/100 g)	< 3	< 3	< 3	< 3	< 3	< 3	< 3	< 3	< 3	< 3
P (mg/100 g wet weight)	188.3 ± 6.6^d^	162.6 ± 5.5^b^	194.5 ± 8.7^e^	181.7 ± 7.6^c^	143.1 ± 6.5^a^	176.4 ± 5.3^c^	178.3 ± 5.8^c^	218.2 ± 6.4^f^	238.6 ± 8.0^h^	224.0 ± 7.5^g^
K (mg/100 g wet weight)	1.81 ± 0.05^c^	1.84 ± 0.05^c^	1.99 ± 0.04^e^	1.26 ± 0.04^b^	1.22 ± 0.05^b^	1.81 ± 0.07^c^	1.92 ± 0.06^d^	1.05 ± 0.04^a^	2.06 ± 0.07^f^	2.17 ± 0.06^g^
Mg (mg/Kg wet weight)	119.8 ± 5.5^e^	101.2 ± 4.4^c^	98.3 ± 5.6^c^	112.4 ± 5.8^e^	124.0 ± 6.7^d^	92.4 ± 4.5^b^	73.8 ± 4.2^a^	71.5 ± 4.9^a^	97.1 ± 5.3^bc^	140.2 ± 6.6^f^
Ca (mg/Kg wet weight)	268.3 ± 1.2^c^	194.2 ± 6.9^a^	214.5 ± 9.4^b^	446.9 ± 17.0^g^	288.4 ± 11.2^d^	220.7 ± 10.1^b^	214.6 ± 9.3^b^	364.6 ± 15.6^f^	210.3 ± 8.5^b^	305.4 ± 13.2^e^
Fe (mg/Kg wet weight)	< 3	< 3	< 3	< 3	< 3	< 3	< 3	< 3	< 3	< 3
Zn (mg/Kg wet weight)	< 2	< 2	< 2	< 2	< 2	< 2	< 2	< 2	< 2	< 2
Cu (mg/Kg wet weight)	0.51 ± 0.04^c^	0.47 ± 0.02^bc^	0.51 ± 0.04^c^	0.34 ± 0.03^a^	0.36 ± 0.02^a^	0.59 ± 0.04^d^	0.45 ± 0.03^b^	0.37 ± 0.02^a^	0.46 ± 0.03^b^	0.77 ± 0.05^e^
Se (mg/Kg wet weight)	<0.03	<0.03	<0.03	<0.03	<0.03	<0.03	<0.03	<0.03	<0.03	<0.03
Mn (mg/Kg wet weight)	0.50 ± 0.03^c^	0.63 ± 0.03^d^	0.43 ± 0.04^b^	0.75 ± 0.05^e^	0.97 ± 0.04^f^	0.64 ± 0.04^d^	0.68 ± 0.03^d^	0.78 ± 0.05^e^	0.39 ± 0.02^a^	0.41 ± 0.04^ab^
F (mg/Kg wet weight)	0.47 ± 0.02^cd^	0.44 ± 0.04^b^	0.45 ± 0.03^bc^	0.49 ± 0.03^d^	0.47 ± 0.02^bcd^	0.51 ± 0.04^d^	0.30 ± 0.02^a^	0.70 ± 0.05^e^	0.74 ± 0.04^f^	0.87 ± 0.05^g^

Values represent mean ± standard deviation, and statistical analysis was carried out by ANOVA plus *post hoc* Ducan’s test, and statistical significance (*p* < 0.05) was indicated with different letters (a–h). AY, Aiyuan 38; BZH, Buzhihuo; CJ, Chunjian; DY, Daya; MRJ, Mingrijian; PG, Ponkan; QC, Qicheng; STJ, Shatangju; WG, Wogan; YH, Yuanhong.

### 3.3. Elements analysis

Citrus juice is a good source of minerals, such as P, K, Mn, Ca, Mg, F, Cu, etc. The types and contents of elements in different varieties of citrus were shown in Table 2. The contents of Na in the citrus samples were lower than 3 mg/100 g, and the contents of K were determined to be 1.05–1.99 mg/kg. The contents of Ca in the pulp of citrus fruits were determined to be 194.2–446.9 mg/Kg. Compared to other fruits, including pears, apples, bananas, melons, plums, peaches, and mangoes, citrus fruits are a valuable source of Ca, which plays an important role in building strong bones (25). The contents of P in the pulp of citrus fruits were determined to be approximately 143.1–218.2 mg/kg. The contents of Mg in citrus samples were determined to be 71.5–124.0 mg/kg. Furthermore, the contents of Cu, Mn, and F in citrus samples were determined to be 0.34–0.59 mg/kg, 0.43–0.97 mg/kg, and 0.30–0.70 mg/kg, respectively. Among the analyzed citrus fruits, significantly higher P and F element contents were found in CJ, the highest Mg and Mn elements were determined in QC, and the highest Ca content was evaluated in PG. Furthermore, AY and MRJ were endowed with the highest K element and Cu element, respectively. Citrus fruits also contain a variety of trace elements, including Fe, Zn, and Se. These essential phytonutrients played important roles in various enzymatic reactions, but their contents were not shown in this study due to low levels. In conclusion, the citrus fruit variety had a significant effect on the content of mineral elements in citrus pulp. This result might attribute to the mineral composition of the soil in which they are grown, the types and amounts of fertilizers used, weather conditions, and the composition of irrigation water (25).

### 3.4. HPLC analysis of phenolic acids and flavonoids

**Figure 2:**
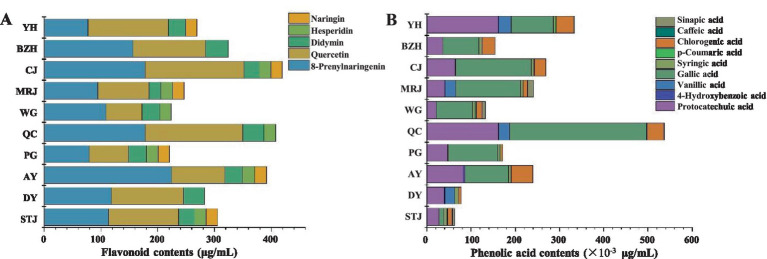
Composition and content of flavonoids **(A)** and phenolic acids **(B)** in different varieties of citrus juice. AY, Aiyuan 38; BZH, Buzhihuo; CJ, Chunjian; DY, Daya; MRJ, Mingrijian; PG, Ponkan; QC, Qicheng; STJ, Shatangju; WG, Wogan; YH, Yuanhong.

The compositions and contents of flavonoids and phenolic acids in 10 different citrus varieties were evaluated. The compositions and contents of flavonoids in 10 different citrus varieties were presented in Figures 2A,B showed the compositions and contents of phenolic acids. The contents of total flavonoids in citrus juice were 220.96–420.35 μg/mL. Statistically, there were significant differences in the content of total phenols acid and total flavonoids among the 10 citrus cultivars. As shown in Figure 2A, the order of total flavonoids content of the 10 citrus varieties was: CJ > QC > AY > BZH > STJ > DY > YH > MRJ > WG > PG. Hesperidin and naringin were identified as the most abundant flavonoids in citrus juice, followed by didymin, quercetin, and 8-prenylnaringenin. Similar results were also reported about the composition of major flavonoids in citrus fruits (26, 27). The highest hesperidin content (175.76 μg/mL) was detected in CJ, and the highest naringin content (222.81 μg/mL) was detected in AY. Moreover, 8-Prenylnaringenin was found in some citrus samples. 8-Prenylnaringenin was a metabolite of xanthohumol mainly found in the *Citrus* genus of plants. A previous study showed that 8-Prenylnaringenin had good anti-cancer, antioxidant, and anti-inflammatory activity, as well as a protective effect on menopausal and post-menopausal symptoms (28). Additionally, the highest content (420.35 μg/mL) of total flavonoids was found in CJ. In 1999, CJ (*Citrus reticulata* × *C. sinensis* cv. Okitsu No.44) was introduced from Japan. CJ has been promoted and cultivated in Jiangxi, Fujian, Sichuan, Chongqing, Hunan, Zhejiang, and other regions of China, and has become one of the citrus varieties with the largest planting area in Sichuan.

Furthermore, phenolic acids were identified and quantified in 10 different citrus juice, including six hydroxybenzoic acids (protocatechuic acid, 4-hydroxybenzoic acid, gallic acid, vanillic acid, salicylic acid, and syringic acid) and four hydroxycinnamic acids (caffeic acid, sinapic acid, chlorogenic acid, and p-coumaric acid). As shown in Figure 2B, protocatechuic acid, gallic acid, and chlorogenic acid were the main phenolic acids detected in all citrus juice, but salicylic acid was not detected in all samples tested. The order of total phenolic acid content of the 10 citrus varieties was: QC > YH > CJ > MRJ > AY > PG > BZH > WG > DY > STJ. The total content of quantified phenolic compounds in different citrus juices ranges from 62.57 to 537.94 × 10^−3^ μg/mL, which might be affected by the species, growing season, environmental factors, ripening, and changes in the storage process (29). It should be noted that QC had the highest content of protocatechuic acid (161.70 × 10^−3^ μg/mL), gallic acid (310.88 × 10^−3^ μg/mL), and total phenolic acids (537.94 × 10^−3^ μg/mL) compared with other varieties of samples. This might be related to the high TA value of QC. Studies suggested that phenolic compounds were important source of antioxidants in citrus juices, but could also be contributed to the blurred appearance and sour taste in fruit juices, resulting in differences in the appearance and taste of the juice (30).

### 3.5. Antioxidant activities

**Figure 3:**
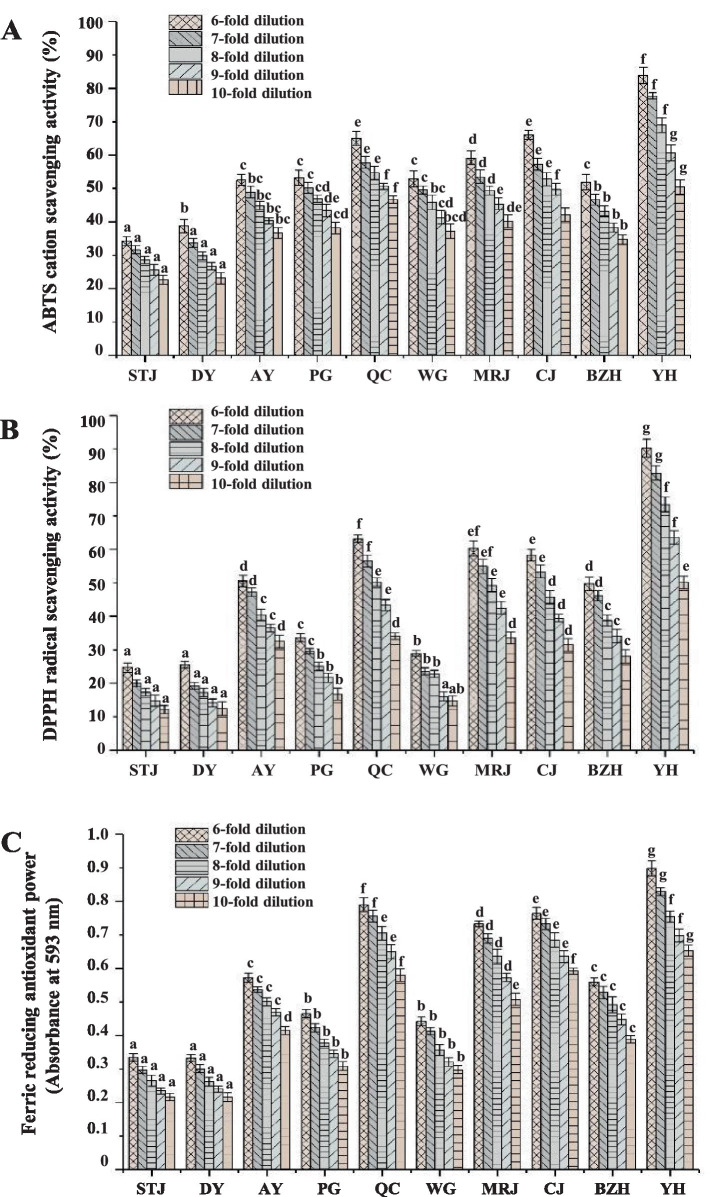
ABTS radical scavenging activity **(A)**, DPPH radical scavenging activity **(B)**, and ferric reducing antioxidant power **(C)** from different varieties of citrus juices. The error bars indicated standard deviation, and statistical analysis was carried out by ANOVA plus *post hoc* Ducan’s test, and statistical significance (*p* < 0.05) was indicated with different lowercase letters (a–g). AY, Aiyuan 38; BZH, Buzhihuo; CJ, Chunjian; DY, Daya; MRJ, Mingrijian; PG, Ponkan; QC, Qicheng; STJ, Shatangju; WG, Wogan; YH, Yuanhong.

The overall antioxidant properties of citrus juice at different dilutions were also evaluated, and the ferric reducing antioxidant power, ABTS cation radical, and DPPH radical scavenging activity of citrus juice at different dilutions were shown in Figure 3. The evaluation of antioxidant activity showed that all the juices examined had significant antioxidant capacities in a dose-dependent manner. Under the same determination method and dilution factor (juice: ultrapure water = 1:5, *v/v*), the results of ABTS cation and DPPH radical scavenging activities for citrus juices from different varieties ranged from 34.25 to 83.82% and 24.71 to 90.17%, respectively. At concentrations of 0.2, 0.4, 0.6, 0.8, and 1.0 mM, the ABTS cation and DPPH radical scavenging activities of the positive controls (Trolox) were determined to be 30.38–77.68 and 16.76–77.02%, respectively. Moreover, all citrus juice samples showed significant ferric-reducing antioxidant power. The FRAP values of citrus juice samples were determined to be 0.334 to 0.898 at the same dilution (juice: ultrapure water = 1:5, *v/v*). By comparison, the absorbance values of the positive control (Trolox) were determined to be 0.366, 0.523, 0.661, 0.849, and 0.984 at concentrations of 0.2, 0.4, 0.6, 0.8, and 1.0 mM, respectively. Overall, all citrus juices exhibited significant antioxidant capacities. YH, QC, and CJ with relatively higher ascorbic acid content, higher flavonoid content, and higher total phenolic content also showed excellent antioxidant activities. The results suggested that the high antioxidant activity of citrus juice might be associated with the content of ascorbic acid, phenolic acids, and flavonoids, which was consistent with the report by Sicari et al. (10).

Interestingly, YH achieved the highest antioxidant capacities (DPPH, ABTS, and FRAP), but it did not show the highest phenolic acid and flavonoid content among the 10 selected citrus juices. After a 10-fold dilution (juice: ultrapure water = 1:9, *v/v*) of freshly squeezed citrus juice, the DPPH, ABTS cation radical scavenging activity, and FRAP value of YH were determined to be 50.43, 50.14%, and 0.653, respectively. YH was a new variety in recent years. It was a hybrid of Moro blood orange and Ota ponkan. YH not only had the shape and sweetness of ponkan but also had the color of blood orange. Numerous studies demonstrated that certain red-colored citrus varieties accumulate anthocyanins, carotenoids, and other components during growth, which imparted a distinctive purple-red coloration to their fruit and juice (31, 32). The possible presence of these components in YH might also contribute to the antioxidant activity of citrus juice. All in all, the results suggested that YH was a valuable variety with excellent antioxidant activity, which might benefit human health. Regrettably, the cultivation time of YH in Sichuan was short and the cultivation technology was still immature, it was necessary to be further promoted and cultivated.

### 3.6. Data analysis

**Figure 4:**
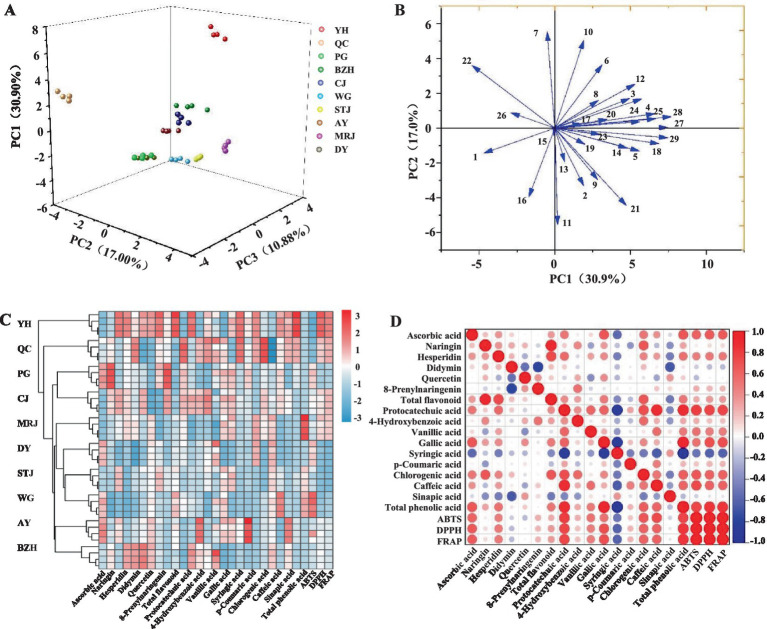
Principal component analysis **(A)**, correlation of variables with PC1 and PC2 **(B)**, hierarchically clustered heat map of different citrus varieties **(C)**, and correlation heatmap between the antioxidant capacity of fruit juice and the composition of antioxidants in fruit juice **(D)**. AY, Aiyuan 38; BZH, Buzhihuo; CJ, Chunjian; DY, Daya; MRJ, Mingrijian; PG, Ponkan; QC, Qicheng; STJ, Shatangju; WG, Wogan; YH, Yuanhong. Numbers 1–29 in Figure 4B correspond to the parameters in Table 2, respectively.

The PCA analysis selected six principal components with eigenvalues above 1 among the 29 tested parameters, including nutritional composition, mineral elements, functional ingredients, and antioxidant tests. The cumulative percent variance (CPV) of the six principal variables was calculated to be 85.90% of the total variance. The first principal component (PC1) explained about 30.90% of the total variance and integrated the content of most nutrients (fat, carbohydrate, dietary fiber, and ascorbic acid), minerals (P, Cu, Ca, F, Mg, and Mn), flavonoids, phenolic acids, as well as antioxidant tests (Table 2). The PC2, PC3, PC4, PC5 and PC6, respectively, explained about 17.00, 10.88, 9.60, 8.87, and 8.65% of the variance. The PCA score plot (Figure 4A) was applied to illustrate whether the citrus investigated could be grouped by variety. The scatter plot of the score values attributable to PC1, PC2 and PC3 clearly showed the differences between the different varieties of citrus based on the 29 tested parameters. In particular, YH and QC showed the most significant differences from other citrus varieties.

Figure 4B projects all 29 tested parameters onto the two-dimensional factor space of the two-dimensional circle. The direction of the arrows explained the correlation between the variables (33). Briefly, the correlation between variables was negatively correlated when two lines pointed in opposite directions, uncorrelated when they were orthogonal, and highly correlated when they were pointing in the same direction. Among the 29 tested parameters, there was an obvious positive correlation between antioxidant parameters (DPPH, ABTS, and FRAP), while no obvious regularity was found among other variables. In addition, all the 29 tested parameters were used to construct a hierarchically clustered heat map among the 10 citrus varieties, as shown in Figure 4C. It could be seen that the hierarchically clustered heat map divided the 10 citrus varieties into different groups, and the difference between YH and the other groups was especially obvious. The results showed that there were significant differences among the 10 citrus varieties, which might be due to the type and content of nutrients, bioactive compounds, and antioxidant activities in citrus fruits, which could be explained by not only the cultivar but also the differences of fertilization, climate, soil type, and origin place.

Correlation analysis revealed an important role of ascorbic acid and phenolic compounds. The heatmap analysis of the correlation between antioxidant capacities was shown in Figure 4D. Based on these results, there was a strong positive correlation among antioxidant activities (ABTS, DPPH, and FRAP), ascorbic acid, and total phenolic acid content. A strong positive relationship was also detected between FRAP and ascorbic acid (*r* = 0.6411), protocatechuic acid (*r* = 0.7579), gallic acid (*r* = 0.7020), chlorogenic acid (*r* = 0.6116), caffeic acid (*r* = 0.6083), and total phenolic acid content (*r* = 0.8297). However, no significant correlation was detected between flavonoids and antioxidant capacity. A previous study reported that citrus fruits were rich in phenolic acids, and the significant contribution of phenolic compounds to antioxidant capacity was identified (27). In addition, Samira and Khodir (34) also found a strong correlation between phenolic acid and antioxidant activity, i.e., the higher the total phenolic content, the more significant the antioxidant activity. Similar results were also reported that citrus fruits, such as limes, lemons, and oranges, exhibited significant antioxidant activity, which was mainly attributed to the ascorbic acid and phenolic compounds (35). It was reported that the strong antioxidant properties of citrus phenolic acids were attributed to the effect of ortho-substitution on the benzene ring and the dehydrogenation of hydroxyl groups (36). Among the citrus hesperidin, flavonoids, naringenin, and naringin had been reported to show significant antioxidant effects (12, 37). Flavonoids inhibit the generation of free radicals, counteracting lipid oxidation, and improve the body’s antioxidant enzyme activity to reduce the formation of peroxides *in vivo* (36). However, flavonoids did not appear to be the principal contributors to the antioxidant activity of citrus juice based on the results of this study.

## 4. Conclusion

In this study, 10 typical citrus varieties from the main cultivated varieties in Sichuan, China were selected, and their quality attributes, juice properties, nutrients, functional components, and antioxidant properties were systematically evaluated and analyzed. The results showed that there were significant differences in quality attributes, nutrients, and functional components among different citrus varieties, which might be affected by the species, origin place, growing season, environmental factors, ripening, and changes in the storage process. Generally, the total soluble sugar content of MRJ produced in Meishan, Sichuan in this study was higher than those of other citrus varieties, suggesting its potential for fresh consumption and food industrial processing. YH with higher total phenolic content, higher flavonoid content, and higher ascorbic acid content was considered to be a valuable variety with excellent antioxidant capacity, and had the potential for further promotion and cultivation. Furthermore, principal component analysis and hierarchical cluster analysis suggested that there were significant differences among the 10 citrus varieties. Correlation analysis revealed the significant contribution of ascorbic acid and phenolic compounds to antioxidant capacity. The results of this study will provide valuable guidance for the identification and utilization of citrus.

## Data availability statement

The original contributions presented in the study are included in the article/supplementary material, further inquiries can be directed to the corresponding authors.

## Author contributions

HGu and R-YG: conceptualization and methodology. HGu: software and writing – original draft. Y-JZ and H-YL: validation. Y-JZ: formal analysis. HGu and H-YL: investigation. Y-JZ, XD, and D-GZ: resources. HGu and H-YL: data curation. HGa, MA, H-BL, H-YL, and R-YG: writing – review and editing. H-YL and R-YG: supervision. Y-JZ, D-TW and R-YG: funding acquisition. All authors have read and agreed to the published version of the manuscript.

## Funding

This work was supported by the Agricultural Science and Technology Innovation Program (ASTIP-IUA-2023003), the Local Financial Funds of the National Agricultural Science and Technology Center, Chengdu (no. NASC2021PC03), the Local Financial Funds of the National Agricultural Science and Technology Center, Chengdu (no. NASC2021KR01), and the Key Scientific Research Fund Project of Science and Technology Department of Sichuan Province (no. 2023YFN0011). Central Public-interest Scientific Institution Basal Research Fund (no. S2023006).

## Conflict of interest

D-GZ was employed by the Pujiang Yuanxiang Modern Agriculture Limited Company.

The remaining authors declare that the research was conducted in the absence of any commercial or financial relationships that could be construed as a potential conflict of interest.

## Publisher’s note

All claims expressed in this article are solely those of the authors and do not necessarily represent those of their affiliated organizations, or those of the publisher, the editors and the reviewers. Any product that may be evaluated in this article, or claim that may be made by its manufacturer, is not guaranteed or endorsed by the publisher.

## Footnotes

^1^ http://www.stats.gov.cn/sj/ndsj/2022/indexch.htm
